# Value of soluble TREM-1, procalcitonin, and C-reactive protein serum levels as biomarkers for detecting bacteremia among sepsis patients with new fever in intensive care units: a prospective cohort study

**DOI:** 10.1186/1471-2334-12-157

**Published:** 2012-07-18

**Authors:** Longxiang Su, Bingchao Han, Changting Liu, Liling Liang, Zhaoxu Jiang, Jie Deng, Peng Yan, Yanhong Jia, Dan Feng, Lixin Xie

**Affiliations:** 1 Department of Respiratory Medicine, Hainan Branch of Chinese PLA General Hospital, Sanya, Hainan Province, 572013, China; 2 Medical College, Nankai University, 94 Weijin Rd, Tianjin, 300071, China; 3 Department of Respiratory Diseases, Chinese PLA General Hospital, Beijing, 100853, China; 4 Department of Respiratory Diseases of South-building, Chinese PLA General Hospital, 28 Fuxing Rd, Beijing, 100853, China; 5 Department of Medical Statistics, Chinese PLA General Hospital, 28 Fuxing Rd, Beijing, 100853, China

**Keywords:** Soluble triggering receptor expressed on myeloid cells 1 (sTREM-1), Fever, Sepsis, Bacteremia, Diagnosis, Prognosis

## Abstract

**Background:**

The purpose of this study was to explore the diagnostic value of soluble triggering receptor expressed on myeloid cells 1 (sTREM-1), procalcitonin (PCT), and C-reactive protein (CRP) serum levels for differentiating sepsis from SIRS, identifying new fever caused by bacteremia, and assessing prognosis when new fever occurred.

**Methods:**

We enrolled 144 intensive care unit (ICU) patients: 60 with systemic inflammatory response syndrome (SIRS) and 84 with sepsis complicated by new fever at more than 48 h after ICU admission. Serum sTREM-1, PCT, and CRP levels were measured on the day of admission and at the occurrence of new fever (>38.3°C) during hospitalization. Based on the blood culture results, the patients were divided into a blood culture-positive bacteremia group (33 patients) and blood culture-negative group (51 patients). Based on 28-day survival, all patients, both blood culture-positive and -negative, were further divided into survivor and nonsurvivor groups.

**Results:**

On ICU day 1, the sepsis group had higher serum sTREM-1, PCT, and CRP levels compared with the SIRS group (*P* <0.05). The areas under the curve (AUC) for these indicators were 0.868 (95% CI, 0.798–0.938), 0.729 (95% CI, 0.637–0.821), and 0.679 (95% CI, 0.578–0.771), respectively. With 108.9 pg/ml as the cut-off point for serum sTREM-1, sensitivity was 0.83 and specificity was 0.81. There was no statistically significant difference in serum sTREM-1 or PCT levels between the blood culture-positive and -negative bacteremia groups with ICU-acquired new fever. However, the nonsurvivors in the blood culture-positive bacteremia group had higher levels of serum sTREM-1 and PCT (*P* <0.05), with a prognostic AUC for serum sTREM-1 of 0.868 (95% CI, 0.740–0.997).

**Conclusions:**

Serum sTREM-1, PCT, and CRP levels each have a role in the early diagnosis of sepsis. Serum sTREM-1, with the highest sensitivity and specificity of all indicators studied, is especially notable. sTREM-1, PCT, and CRP levels are of no use in determining new fever caused by bacteremia in ICU patients, but sTREM-1 levels reflect the prognosis of bacteremia.

**Trial registration:**

ClinicalTrial.gov identifier NCT01410578

## Background

Sepsis is one of the main causes of morbidity and mortality in the intensive care unit (ICU). In the United States, approximately 500,000 people die of sepsis each year [1,2]. Because of the prevalence of patients with fever, leukocytosis, and increased heart and respiratory rates in the ICU as well as the lack of sensitivity and specificity of these parameters, it has been a challenge for physicians to diagnose sepsis by these criteria alone [3,4]. Blood culture has always been held as the gold standard for sepsis diagnosis [5]. However, treatments are normally delayed while waiting for lab results. Therefore, it has been a difficult task for ICU physicians to accurately differentiate bacteremia from similar diseases and come to a rapid diagnosis. In addition, clinical studies have shown that early intervention can significantly improve the prognosis of severe sepsis and septic shock and lower the mortality rate [6,7]. Thus, it becomes critical to identify any biomarker that can accurately identify infection or noninfection as well as quickly diagnose bacteremia and predict its prognosis. In this study, we evaluated the value of soluble triggering receptor expressed on myeloid cells 1 (sTREM-1), procalcitonin (PCT), and C-reactive protein (CRP) serum levels in terms of their value for sepsis diagnosis, identification of new fever caused by bacteremia in ICU patients, and prediction of prognosis.

## Methods

The study was performed from September 2009 to March 2011 in the Respiratory ICU, Surgical ICU, and Emergency ICU of the Chinese People’s Liberation Army (CPLA) General Hospital. This study was approved by the Ethics Committee of the CPLA General Hospital (project No. 20090923–001) and was registered with the U.S. National Institutes of Health Clinical Trials Registry (NCT01410578). Patients or their family members were fully informed and signed informed consent forms.

### Inclusion and exclusion criteria

Based on the 1991 ACCP/SCCM Sepsis Directory [8] and the diagnostic criteria advanced by the 2001 International Sepsis Definition Conference [9], patients exhibiting two or more of the following signs during their first 24 h in the ICU were eligible for selection: (1) temperature of >38°C or <36°C, (2) pulse rate of >90 beats/min, (3) respiratory rate of >20 breaths/min or hyperventilation with a partial pressure of arterial carbon dioxide (PaCO_2_) of <32 mmHg, or (4) white blood cell (WBC) count of >12,000μL^-1^ or <4000 μL^-1^, or >10% immature cells. Exclusion criteria were: (1) <18 years of age, (2) acquired immunodeficiency syndrome, (3) reduced polymorphonuclear granulocyte count (<500 μL^-1^), or (4) died within 24 h after admission into the ICU, refused to participate in the study, or declined treatment during the observation period.

### Infection and bacteremia definition

The presence of infection, defined according to the clinical and microbiological criteria of the CDC definitions [10,11], was held as a gold standard and determined by three independent experts who were blinded to the CRP, PCT, and WBC results [12]. Blood culture-positive bacteremia was defined as growth of bacteria with recognized pathogenic capacity in one blood culture or as growth of common skin pathogens (i.e., coagulase-negative *Staphylococcus* species, diphtheroids, *Bacillus* species, *Propionibacterium* species, or micrococci) in two blood cultures [13].

### New fever in ICUs

Fever is a common problem in ICU patients and is mainly traced to multiple infectious or noninfectious causes [14]. The definition of fever is arbitrary and depends on the purpose for which it is defined. Guidelines for evaluation of new fever in critically ill adult patients define fever in the ICU as a temperature of >38.3°C [15,16]. Taking blood cultures from the patient is the initial step in addressing fever [17]. In this study, “new fever” refers to a new fever (temperature >38.3°C) that occurred more than 48 h after ICU admission.

### Clinical data and sample collection

Demographic and disease data of the patients admitted to the ICU included age, gender, chief complaint upon admission, major diagnosis, clinical manifestation, Acute Physiology and Chronic Health Evaluation II (APACHE II) score, Sequential Organ Failure Assessment (SOFA) score, WBC counts, CRP levels, PCT levels, sTREM-1 levels, cause of infection, initial disease status, and survival during the first 28 days. When the patients were admitted to the ICU, 3 mL of intravenous blood was collected within the first 24 h. The blood was centrifuged at 3000 rpm/min at 4°C for 15 min. Samples were collected again if the patient’s temperature reached >38.3°C more than 48 h after admission [14,17]. According to the blood culture standards of the Clinical Laboratory Standards Institute (CLSI) [18], two sets of blood samples were collected from two different sites and were inoculated separately into a Bact/ALERT aerobic bottle and a Bact/ALERT anaerobic bottle (bioMérieux, Marcy-l'Etoile, France).

### Serological assays and blood culture procedure

All specimens were renumbered before the experiment. The researchers were relatively blind to each step. sTREM-1 levels were determined in duplicate using a double antibody sandwich enzyme-linked immunosorbent assay (ELISA) (Quantikine Human TREM-1 Immunoassay kit; R&D Systems, Minneapolis, MN, USA). PCT was measured using enzyme-linked fluorescence analysis (ELFA) (VIDAS BRAHMS PCT kit; bioMérieux). CRP was determined using scattering turbidimetry (CardioPhase hsCRP; Siemens Healthcare Diagnostics, Deerfield, IL, USA). A BacT/ALERT 3D automation system (bioMérieux) was used to continuously monitor the blood samples in the blood culture bottles. As soon as bacterial growth was detected, the positive bottle was sent for single-colony subculture. Pathogens were identified manually or using the VITEK II system, and bacterial contaminants were excluded [19,20]. All procedures were strictly conducted following the manufacturers’ instructions and standard microbiology guidelines.

### Statistical analysis

SPSS statistical software v16.0 (SPSS, Chicago, IL, USA) was used for data analysis. The normally distributed variances were expressed as the mean ± SD. Student’s t-test was used to compare mean values between two groups. The data that were not normally distributed were expressed as medium (interquartile ranges) and analyzed using the rank sum test. Unordered categorical variables were expressed as percentages, and the difference in proportion between two groups was analyzed using the chi-square test. A receiver operating characteristic (ROC) curve was employed to evaluate the effects of sTREM-1, PCT, and CRP levels on sepsis diagnosis, new fever caused by bacteremia, and the prognosis of patients with bacteremia.

## Results

### Patient characteristics

**Figure 1:**
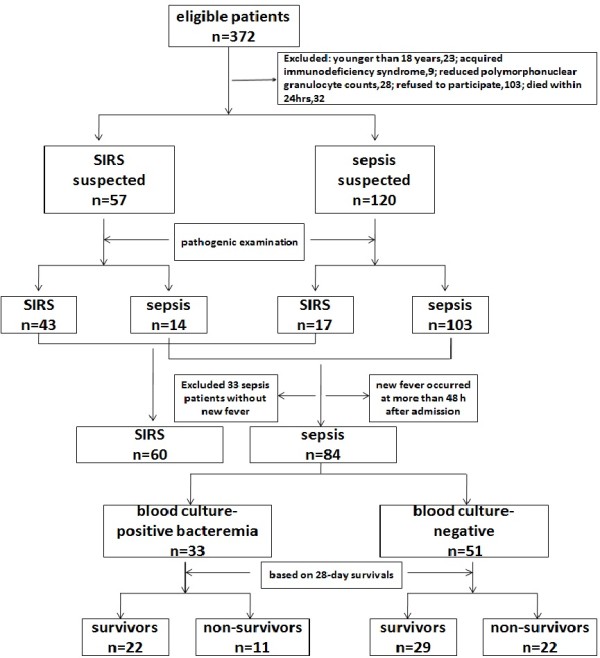
Profiles of patients enrolled in our study

A total of 372 patients were eligible for this study. Of these, 177 were admitted to the ICU within 24 h with systemic inflammatory response syndrome (SIRS) criteria, and 195 were excluded. The 177 patients were divided into 57 with suspected SIRS and 120 with suspected sepsis. According to microbial culture results, these patients were further classified into two subgroups: those with SIRS (n = 60) and those with sepsis complicated with new fever (n = 84). A total of 84 patients with sepsis were placed into either a blood culture-positive bacteremia group (n = 33) or a blood culture-negative group (n = 51) based on blood culture tests at the occurrence of new fever. Based on 28-day survivals, patients with blood culture-positive bacteremia were further divided into a survivor group (n = 22) and a nonsurvivor group (n = 11), as were the patients with a negative blood culture (29 survivors and 22 nonsurvivors). A flow chart is shown in Figure 1.

The patients’ baseline data at admission are shown in Table 1. Temperatures and APACHE II and SOFA scores were notably higher in the sepsis group than in the SIRS group (*P* <0.001); however, the initial temperature and APACHE II and SOFA scores demonstrated no correlation with blood culture-positive status. In terms of the source of infection, the percentage of catheter-related infections was considerably higher in the blood culture-positive group than in the blood culture-negative group (*P* = 0.006). However, pulmonary, abdominal, urinary tract, and trauma/postoperative infections were not significantly different between these two groups. In terms of predisposing factors, the rate of immunosuppression was higher in the sepsis group than in the SIRS group (*P* = 0.011). Conversely, the presence or absence of immunosuppression in the initial stage did not correlate with positivity of blood culture. Furthermore, there was no significant difference in any of the other predisposing factors among the three groups (SIRS, sepsis with blood culture-positive bacteremia, and sepsis with a negative blood culture). Mortality rate was much higher in the sepsis group than in the SIRS group (*P* < 0.001), but this did not correlate with blood culture positivity. No statistical difference was noted for age or gender among the three groups.

**Table 1:** Clinical and biological data at ICU admission according to SIRS and sepsis

**Characteristics**	**SIRS**	**sepsis**		**P value**
		**negative blood culture**	**positive blood culture**	
	**N = 60**	**N = 51**	**N = 33**	
**Age (years)**	51 ± 21	59 ± 18	54 ± 20	**NS**
**Gender (n,%)**				**NS**
Male	36 (60)	31 (60.8)	25 (75.8)	
Female	24 (40)	20 (39.2)	8 (24.2)	
**Temperature (°C)**	37.2 ± 0.9	38.4 ± 0.9	38.0 ± 1.2	**<0.001**
**APACHE II score**	11 ± 7	18 ± 8	17 ± 8	**<0.001**
**SOFA score**	-	8 ± 4	8 ± 4	**NS**
**Etiological factors (n,%)**				
Pulmonary infection	-	40 (78.4)	23 (69.7)	**NS**
Abdominal infection	-	11 (21.6)	6 (18.2)	**NS**
Urinary tract infection	-	10 (19.6)	8 (24.2)	**NS**
Trauma/postoperative infection	-	16 (31.4)	14 (42.4)	**NS**
Catheter-related infections	-	3 (5.9)	9 (27.3)	**0.006**
Others	-	4 (3)	1 (3)	**NS**
**Predisposing factors(n,%)**				
Hypertension	15 (25)	19 (37.3)	10 (30.3)	**NS**
Diabetes	4 (6.7)	10 (19.6)	4 (12.1)	**NS**
COPD	2 (3.3)	6 (11.8)	3 (9.1)	**NS**
Coronary heart disease	7 (11.7)	7 (13.7)	2 (6.1)	**NS**
Immunosuppressed	0 (0)	5 (9.8)	3 (9.1)	**0.011**
Nervous system disease	3 (5)	5 (5.9)	2 (6.1)	**NS**
CKD	2 (3.3)	3 (5.9)	3 (9.1)	**NS**
**Mortality rate (n,%)**	4 (6.7)	22 (43.1)	11 (33.3)	**P <0.001**

NS, not significant. Quantitative data of normal distribution are presented as mean ± SD. Quantitative data of non-normal distribution are presented as median (interquartile range). Qualitative data are presented as n (%).

*APACHE II score* Acute Physiologic Assessment and Chronic Health Evaluation II score; *SOFA score* Sequential Organ Failure Assessment score; *COPD* chronic obstructive pulmonary disease; *CKD* chronic kidney disease.

### sTREM-1, CRP, and PCT: early diagnosis of sepsis on the day of ICU admission

**Figure 2:**
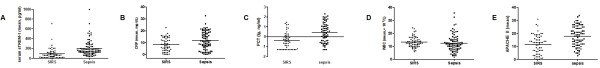
**Comparison of diagnostic values for sepsis.** The diagnostic values of sTREM-1, CRP, and PCT levels; WBC count; and APACHE II score for sepsis on the day ICU admission were compared. The dots denote individual values, and the bars denote means

**Figure 3:**
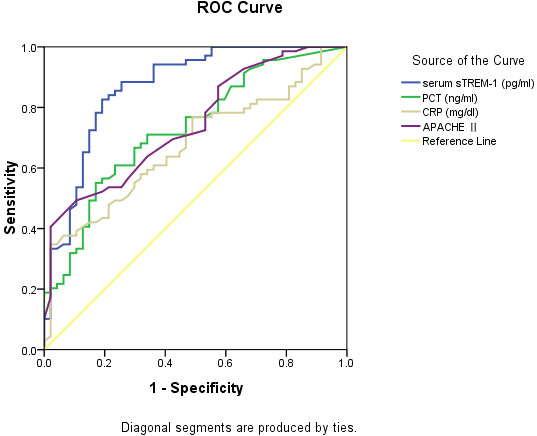
**ROC curves for sepsis versus SIRS.** Shown are the ROC curves for sTREM-1, CRP, and PCT levels and APACHE II score for distinguishing sepsis from SIRS

sTREM-1, CRP, and PCT levels and APACHE II scores on the day of ICU admission are shown in Figure 2. These values were significantly higher in the sepsis group than in the SIRS group (149.06 vs. 59.97 pg/mL, *P* <0.001; 2.86 vs. 0.33 ng/mL, *P* <0.001; 11.4 vs. 8.3 mg/dL, *P* = 0.001; and 18 vs. 11, *P* <0.001, respectively). On the other hand, there was no difference in WBC counts (13.32 vs. 12.54 × 10^9^/L, respectively; *P* = 0.373). ROC curves were obtained for sTREM-1, PCT, and CRP levels and APACHE II scores, which were significantly different between the SIRS and sepsis groups (Figure 3). The areas under the curve (AUC) for sTREM-1, PCT, and CRP levels and APACHE II scores were 0.868 (95% CI, 0.798–0.938), 0.729 (95% CI, 0.637–0.821), 0.679 (95% CI, 0.578–0.771), and 0.745 (95% CI, 0.656–0.833), respectively. When 108.9 pg/mL was set as the cut-off value for sTREM-1, the sensitivity was 0.83 and specificity was 0.81 (Table 2).

**Table 2:** Area under ROC curve as a means of differentiating sepsis from SIRS

**variable**	**AUC**	**Std. Error**	**P value**	**Asymptotic 95% Confidence Interval**		**Cut**	**sen**	**spe**	**PPV**	**NPV**	**PLR**	**NLR**	**YI**
				**Lower limit**	**Upper limit**	**point**							
**sTREM-1**	0.868	0.036	<0.001	0.798	0.938	108.9	0.83	0.81	0.86	0.65	2.9	0.31	0.64
**PCT**	0.729	0.047	<0.001	0.637	0.821	2.1	0.55	0.83	0.82	0.42	2.14	0.51	0.38
**CRP**	0.675	0.049	0.001	0.578	0.771	16.5	0.35	0.98	0.96	0.35	2.85	0.76	0.33
**APACHEII score**	0.745	0.045	<0.001	0.656	0.833	19.5	0.49	0.89	0.86	0.4	3.49	0.62	0.39

*PPV* Positive Predictive Value; *NPV* Negtive Predictive Value; *PLR* Positive Likelihood Ratio; *NLR* Negative Likelihood Ratio; *YI* Youden Index.

### sTREM-1, PCT, and CRP: bacteremia in patients with sepsis and new fever

**Figure 4:**
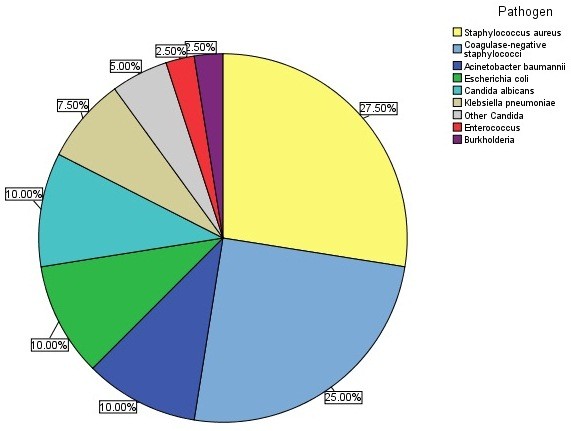
**Causes of bacteremia.** Shown are the relative proportions of causes of bacteremia from 33 positive blood cultures

**Figure 5:**
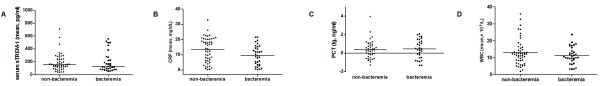
**Comparison of diagnostic values for bacteremia.** The diagnostic values of sTREM-1, CRP, and PCT levels and WBC count for bacteremia were compared. The dots denote individual values, and the bars denote means

Eighty-four patients with sepsis had new fever (>38.3°C) during their ICU stay (>48 h after ICU admission). Thirty-three samples were positive for bacteremia (39.2%). The causes of bacteremia are shown in Figure 4. Among the 33 bacteremia-positive samples, 40 different pathogens were discovered through culturing, including 22 Gram-positive bacteria: *Staphylococcus aureus* (27.5%), coagulase-negative *Staphylococcus* (25%), and *Enterococcus* (2.5%); 12 Gram-negative bacteria: *Acinetobacter baumannii* (10%), *Escherichia coli* (10%), and *Klebsiella pneumoniae* (7.5%); and 4 *Candida albicans* (10%). In addition, seven patients had co-infection of two or more pathogens (17.5%). In a comparison between the nonbacteremia and bacteremia groups, serum sTREM-1, PCT, and WBC counts were 155.43 (123.36) vs. 131.57 (194.81) pg/mL, *P* = 0.724; 2.39 (8.1) vs. 2.71 (25) ng/ml, *P* = 0.693; and 12.98 ± 7.58 vs. 11.3 ± 5.01 × 10^9^/L, *P* = 0.264, respectively. However, a comparison of the CRP level between the two groups showed a statistically significant difference (13.19 ± 8.03 vs. 9.55 ± 6.52 mg/dL, respectively; *P* <0.033) (Figure 5).

### sTREM-1, PCT, and CRP: prognostic value

**Figure 6:**
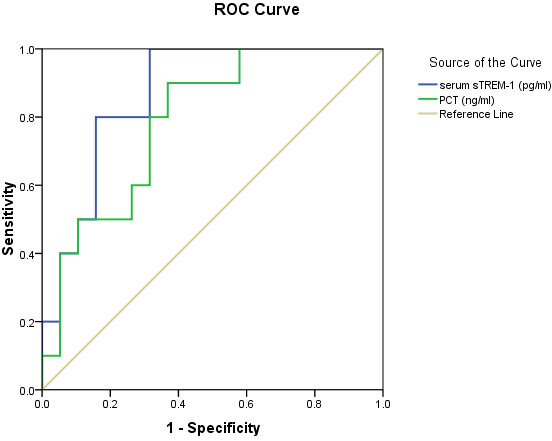
**ROC curves for nonsurvivors versus blood culture-positives.** Shown are the ROC curves for sTREM-1 and PCT for distinguishing nonsurvivors from blood culture-positives

We compared all parameters of the 22 survivors and the 11 nonsurvivors with a blood culture-positive status (Table 3). Body temperature, WBC count, and sTREM-1, PCT, and CRP levels of the nonsurvivors were higher than those of the survivors, although only sTREM-1 and PCT levels between the groups were significantly different. However, in the blood culture-negative group, differences in all of these indicators between the survivors and nonsurvivors were insignificant. Furthermore, ROC curves for the sTREM-1 and PCT levels to predict bacteremia prognosis were determined (Figure 6), and the AUC values for the sTREM-1 and PCT levels were 0.868 (95% CI, 0.740–0.997) and 0.789 (95% CI, 0.624–0.995), respectively. With 126.9 pg/mL as the cut-off value for sTREM-1, the sensitivity and specificity were 1 and 0.68, respectively (Table 4). Multivariable logistic regression was employed to assess possible risk factors for bacteremia. The variables taken into account included serum sTREM-1 and PCT levels. Finally, only serum sTREM-1 was able to enter the regression equation, with a regression coefficient of 0.007, OR of 1.007, Wald coefficient of 5.716, and 95% CI of 1.001 to 1.013 (*P* = 0.017).

**Table 3:** Prognostic value of temperature, WBC, and sTREM-1, CRP, and PCT levels

**Parameter**	**Blood culture-positive bacteremia**		**P value**	**Blood culture-negative**		**P value**
	**survivors**	**non-survivors**		**survivors**	**non-survivors**	
	**n = 22**	**n = 11**		**n = 29**	**n = 22**	
Temperature(mean ± SD,°C)^*^	38.8 ± 0.5	38.9 ± 0.7	**0.559**	39.0 ± 0.5	38.8 ± 0.4	**0.272**
**WBC( mean ± SD, ×10**^**∧**^9/L)^*^	10.8 ± 3.6	12.2 ± 7.2	**0.559**	12.7 ± 8.0	13.4 ± 7.1	**0.747**
**sTREM-1(median-interquartile range, pg/ml)**^**#**^	105.6(90.8)	273.6(350.6)	**0.004**	137.1(159.2)	168.3(150.2)	**0.068**
**PCT (median-interquartile range, ng/ml)**^**#**^	1.2(11.4)	19.9(61.7)	**0.011**	2.6(6.8)	1.8(9.6)	**0.622**
CRP(median-interquartile range, mg/dl)^*^	8.9 ± 7.1	10.9 ± 5.2	**0.408**	12.6 ± 7.9	13.9 ± 8.3	**0.584**

*P-value, Student’s t-test.

#*P*-value, rank sum test.

**Table 4:** Area under ROC curve as a means of differentiating nonsurvivors from blood culture-positives

**variable**	**AUC**	**Std. Error**	**P value**	**Asymptotic 95% Confidence Interval**		**Cut point**	**sen**	**spe**	**PPV**	**NPV**	**PLR**	**NLR**	**YI**
				**Lower limit**	**Upper limit**								
**sTREM-1**	0.868	0.066	0.001	0.74	0.997	126.94	1.0	0.68	0.61	1.00	3.33	0.13	0.68
**PCT**	0.789	0.085	0.012	0.624	0.955	2.26	0.9	0.63	0.55	0.86	2.57	0.27	0.53

*PPV* Positive Predictive Value; *NPV* Negtive Predictive Value; *PLR* Positive Likelihood Ratio; *NLR* Negative Likelihood Ratio; *YI* Youden Index.

## Discussion

TREM-1 is a recently discovered member of the immunoglobulin superfamily of receptors that is specifically expressed on the surfaces of neutrophils and monocytes. sTREM-1 is a soluble form of TREM-1 and is released into bodily fluids when TREM-1 is upregulated. Many studies have indicated that sTREM-1 could be a valuable diagnostic biomarker for various infectious diseases [21-28]. Dynamic changes in sTREM-1 levels could predict survival and mortality of patients at the early stage of sepsis [28-30]. PCT expression is related to the severity of bacterial infection; hence, it is one of the biomarkers for infection [31,32]. PCT measurements along with other clinical tests for infection might be valuable for determining the prognosis of patients [33,34]. CRP is a biomarker involved in variety of inflammatory diseases. Although the majority of hospitals can widely implement CRP analysis in sepsis diagnosis and prognosis [35,36], whether CRP is a good biomarker for early diagnosis of sepsis or bacteremia is still controversial [37,38]. Our study found that sTREM-1, PCT, and CRP levels indicate infection, while sTREM-1 and PCT levels predict prognosis. However, none of these parameters brings to light the cause of new fever. Moreover, sTREM-1 is the best indicator for diagnosis of sepsis and assessment of prognosis of blood culture-positive bacteremia.

Rivera-Chavaz *et al.*[39] performed a study involving 93 patients in the ICU with SIRS symptoms and suspected infection. The patients were classified as having SIRS (no infection; n = 37) or sepsis (n = 56) according to the diagnosis of the physician in charge and clinical evidence. Patients with sepsis had significantly higher sTREM-1 levels than did those with SIRS. At a cut-off of 30 pg/mL, sTREM-1 correctly identified patients suffering from infection with 96% sensitivity and 91% specificity. Porfyridis *et al*. [40] enrolled 68 patients with acute respiratory illness. A total of 34 patients were diagnosed with community-acquired bacterial pneumonia and 34 with nonbacterial pulmonary disease. sTREM-1 levels were significantly higher in the pneumonia group than in the nonbacterial pulmonary disease group, and this analysis was more sensitive and specific than analysis with CRP levels. Yong J *et al.*[41] performed a meta-analysis of 13 clinical studies that fulfilled the inclusion criteria (980 patients; 557 patients with bacterial and 423 with nonbacterial infections). They found that sTREM-1 level for the diagnosis of infection in the AUC of the summary ROC was 0.86, with a sensitivity of 0.82 and specificity of 0.86. This finding confirmed that sTREM-1 is a reliable biomarker for bacterial infection. However, other studies argue that sTREM-1 is of no value for infection diagnosis [42-44]. Some experts have suggested that CRP and PCT levels are more sensitive than sTREM-1 as biomarkers for the diagnosis of bacterial infection [45,46]. Our study, on the other hand, found that on the day of ICU admission, the sepsis group had higher sTREM-1, PCT, and CRP levels and APACHE II scores than did the SIRS group. The indicators above, to different degrees, have values in sepsis identification or diagnosis, of which sTREM-1 proves most efficient. Data obtained through our study are quite similar to those previously reported.

There have been numerous studies on PCT. Stefan *et al.*[47] enrolled 295 patients whose blood culture samples were collected at the emergency department. Based on the blood culture results, the patients were categorized into blood culture-positive, blood culture-negative, and blood culture-contaminated groups. The results indicated that PCT is the most valuable biomarker for the diagnosis of sepsis and bacteremia for patients in the emergency department. Kim *et al.*[48] and Lai *et al*. [38,49] also arrived at the same conclusion: that PCT levels are the most meaningful parameter for bacteremia diagnosis and for patients with bacteremia and a high fever in the emergency department. On the contrary, CRP levels have no value for diagnosis of patients with bacteremia and a high fever in the emergency department. However, Blijlevens *et al.*[50] questioned the value of PCT levels in sepsis diagnosis. Ruiz-Gonzalez *et al.*[51] recently suggested that sTREM-1 levels are valuable for diagnosing bacteremia with community-acquired pneumonia. Because fewer studies have shown the value of sTREM-1 in the diagnosis of bacteremia, we divided ICU patients with fever into blood culture-positive bacteremia and blood culture-negative groups based on their blood culture results. We found that sTREM-1 and PCT levels in the two groups were very comparable. Interestingly, CRP levels were significantly higher in the blood culture-negative group than in the bacteremia group (*P* = 0.033). We speculate that this occurred because of the rise in CRP reactivity rather than because of the bacteremia itself. Our study implies that sTREM-1, CRP, and PCT levels may possess no clinical value in determining whether a given septic patient is complicated with bacteremia on the grounds of a new fever.

Gibot *et al*. [30] studied sTREM-1 levels in 63 patients with severe sepsis and found that decreased sTREM-1 plasma levels were positively correlated with a better prognosis. Thus, sTREM-1 is an excellent biomarker for the prognosis of sepsis. Furthermore, our previous studies and Gibot S *et al*. [28-30] found that compared with PCT and CRP levels, dynamic changes in sTREM-1 levels better predict the prognosis of sepsis. However, no studies have reported whether sTREM-1, PCT, or CRP levels are good parameters for the prognosis of bacteremia. We herein showed that sTREM-1 and PCT levels were useful for the prognosis of blood culture-positive bacteremia. For example, within the bacteremia group, sTREM-1 and PCT levels were significantly higher in nonsurvivors than in survivors. However, we failed to find any prognostic value of sTREM-1 or PCT for blood culture-negative sepsis. Moreover, according to the ROC curves of sTREM-1 and PCT levels, sTREM-1 is a more ideal predictor for the prognosis of blood culture-positive bacteremia. Thus, physicians are expected to pay close attention to patients with high levels of sTREM-1.

Our study was, however, limited by the following factors. (1) The study involved a small sample size; only 33 patients with a positive blood culture were enrolled. (2) Only those who developed a new fever (>38.3°C) in the ICU were enrolled. Therefore, we cannot exclude the possibility that patients who did not have a high fever also had bacteremia; unfortunately, however, no such patients were involved in our analyses. (3) The incidence of opportunistic infections is higher in the ICU, and many pathogens circulate in the wards. Consequently, the possibility of false-positive blood cultures could not be excluded. Furthermore, the patients received regular antibiotic treatments, and side effects of such a long-term treatment should not have been ignored. (4) It is well known that more than two-thirds of patients with severe bacterial sepsis have negative blood cultures [52,53], but this fact (blood culture negative bacteremia) was not properly addressed in the study.

## Conclusions

sTREM-1, PCT, and CRP levels are of substantial value for the early diagnosis of sepsis. In addition, sTREM-1 levels can reflect the infection status more accurately and more specifically than can CRP and PCT levels. However, none of these three parameters could be used to determine whether the fever of patients with sepsis was caused by bacteremia. Finally, sTREM-1 and PCT levels could aid in predicting the prognosis of bacteremic patients. Regrettably, the sample size of this study was small; larger studies are thus needed to further evaluate the value of sTREM-1, PCT, and CRP levels in the diagnosis of bacteremia.

## Abbreviations

sTREM-1, Soluble triggering receptor expressed on myeloid cells 1; PCT, Procalcitonin; CRP, C-reactive protein; ICU, Intensive care unit; SIRS, Systemic inflammatory response syndrome; AUC, Areas under the curve; WBC, White blood cell; APACHE II, Acute Physiology and Chronic Health Evaluation II; SOFA, Sequential Organ Failure Assessment; ROC curve, Receiver operating characteristic curve; COPD, Chronic obstructive pulmonary disease; CKD, Chronic kidney disease; PPV, Positive Predictive Value; NPV, Negtive Predictive Value; PLR, Positive Likelihood Ratio; NLR, Negative Likelihood Ratio; YI, Youden Index.

## Misc

Longxiang Su and Bingchao Han contributed equally to this work

## Competing interests

All authors declare that they have no competing interests.

## Authors’ contributions

LS and CL designed the study, performed the data analysis, and wrote the first manuscript draft. PY, JD, and YJ carried out the study in the SICU, EICU, and RICU, respectively. BH, LL and CJ performed the experiments. DF performed the statistical work. LX was responsible for protocol revisions, data analysis, and final draft revision. All authors have read and approved the final manuscript.

## Pre-publication history

The pre-publication history for this paper can be accessed here:

http://www.biomedcentral.com/1471-2334/12/157/prepub
